# Perceptual mechanisms of social affiliation in zebrafish

**DOI:** 10.1038/s41598-020-60154-8

**Published:** 2020-02-27

**Authors:** Ana Rita Nunes, Leonor Carreira, Savani Anbalagan, Janna Blechman, Gil Levkowitz, Rui F. Oliveira

**Affiliations:** 1 Gulbenkian Institute of Science, Oeiras, Portugal; 2 Weizmann Institute of Science, Rehovot, Israel; 3 ISPA - Instituto Universitário, Lisboa, Portugal; 4 Present Address: ReMedy-International Research Agenda Unit, Centre of New Technologies, University of Warsaw, 02-097 Warsaw, Poland; Laboratory of Glial Biology, Centre of New Technologies, University of Warsaw, 02-097, Warsaw, Poland

**Keywords:** Neuroscience, Psychology, Zoology

## Abstract

Social living animals need to recognize the presence of conspecifics in the environment in order to engage in adaptive social interactions. Social cues can be detected through different sensory modalities, including vision. Two main visual features can convey information about the presence of conspecifics: body form and biological motion (BM). Given the role that oxytocin plays in social behavior regulation across vertebrates, particularly in the salience and reward values of social stimuli, we hypothesized that it may also be involved in the modulation of perceptual mechanisms for conspecific detection. Here, using videoplaybacks, we assessed the role of conspecific form and BM in zebrafish social affiliation, and how oxytocin regulates the perception of these cues. We demonstrated that while each visual cue is important for social attraction, BM promotes a higher fish engagement than the static conspecific form alone. Moreover, using a mutant line for one of the two oxytocin receptors, we show that oxytocin signaling is involved in the regulation of BM detection but not conspecific form recognition. In summary, our results indicate that, apart from oxytocin role in the regulation of social behaviors through its effect on higher-order cognitive mechanisms, it may regulate social behavior by modulating very basic perceptual mechanisms underlying the detection of socially-relevant cues.

## Introduction

For animals to interact with each other, they have to distinguish other individuals from inanimate objects in the environment, and especially to recognize their conspecifics. Social species in particular must have sensory abilities tuned to their social environment and be very efficient at extracting cues about distinctive conspecific features. These cues are detected by specialized sensory channels, processed by specific nuclei in the brain and integrated in specialized neuronal circuits to produce appropriate behavioral responses. Individuals first recognize their conspecifics and then decide whether or not to interact. Thus, these sensory cues are the foundation of social behaviors and crucial for individual survival^1^.

Among the different sensory modalities, vision plays a fundamental role in social affiliation in humans, non-human primates and other species, including fish^2–5^. As an important model in social neuroscience, zebrafish (*Danio rerio*) have a well-characterized repertoire of social behaviors including a robust affiliative behavior^6^, and rely on visual cues to recognize conspecifics. When they are exposed to conspecifics through visual stimuli alone, either real or in videos, zebrafish immediately approach the conspecifics to interact and form shoals^7–9^. Affiliation with a social group is critical for their survival since it facilitates foraging, sexual interactions and predator avoidance^10^. Zebrafish distinguish their conspecifics by their overall appearance and are sensitive to specific traits such as color, shape and striping pattern^2,11,12^. Zebrafish are also sensitive to conspecific motion pattern. For instance, in the presence of fish robots mimicking their visual features, zebrafish display a preference towards robots moving like a conspecific rather than those that are static^13^. Moreover, they also pay attention to the conspecific motion pattern when visual form features are not present. This has first been shown using videos of dyads fighting in which fighting fish were replaced by fighting dots^14^, and more recently, by a single dot mimicking zebrafish motion which was sufficient to promote affiliative behaviors^15^. Although this last work had shown the importance of detection of biological motion (BM) for the expression of affiliative behavior, the contribution of conspecific shape and the underpinning of the molecular mechanisms involved in the perception of BM have not yet been explored.

One potential candidate to modulate BM perception is oxytocin (OXT), a key neuropeptide from the vasotocin nonapeptide family known to regulate social behaviors across species, including zebrafish^16–18^. In fish, isotocin, the ortholog of mammalian OXT only differs from it by 2 amino acids and due to their similarly in both structure and function, it has been recently referred as OXT in the fish literature (https://zfin.org/ZDB-GENE-030407-1)^19,20^.

OXT has been implicated in the perception of social cues by increasing the salience and rewarding value of social stimuli^21–23^. This neuromodulator acts by binding to oxytocin receptors (OXTR) expressed in specific brain nuclei that are part of a conserved Social Decision-Making Network, known to regulate social behaviors across species^18,24^. OXTR are also expressed in sensory processing areas of the brain, and thus, OXT may also be involved in the perception of social cues. In rodents, a taxon that uses predominantly olfaction in conspecific recognition, OXTR is highly expressed in the anterior olfactory nucleus^25,26^. In monkeys and teleost fish, both taxa relying on visual cues for conspecific recognition, OXTR are expressed in the subcortical and early cortical visual areas, and optic tectum, respectively^27,28^. In zebrafish, two different oxytocin receptors have been identified (oxtr gene NM_001199370.1 and oxtr1-like gene NM_001199369)^29^.

It has been shown that OXT is involved in the perception of BM in both humans and dogs, using a point-light display paradigm simulating their motion, that not only includes information about the movement itself but also form or contours from the global configuration of the points used^30,31^. Furthermore, impairments in either BM visual perception and/or OXT circuits have been associated with social deficits in neurological disorders, but their underlying mechanisms are still not well understood^32–34^.

The aim of the present work is to investigate how zebrafish integrates two main social visual features, biological form and motion, to guide social behaviors, and to assess whether OXT is involved in regulating the perception of these stimuli. For this purpose, we used a video playback system of computer animations that allowed us to disentangle the effects of conspecific form from those of BM in the zebrafish affiliative response towards conspecifics.

## Results

### Biological motion and conspecific form are sufficient for zebrafish attraction to visual social stimuli

**Figure 1:**
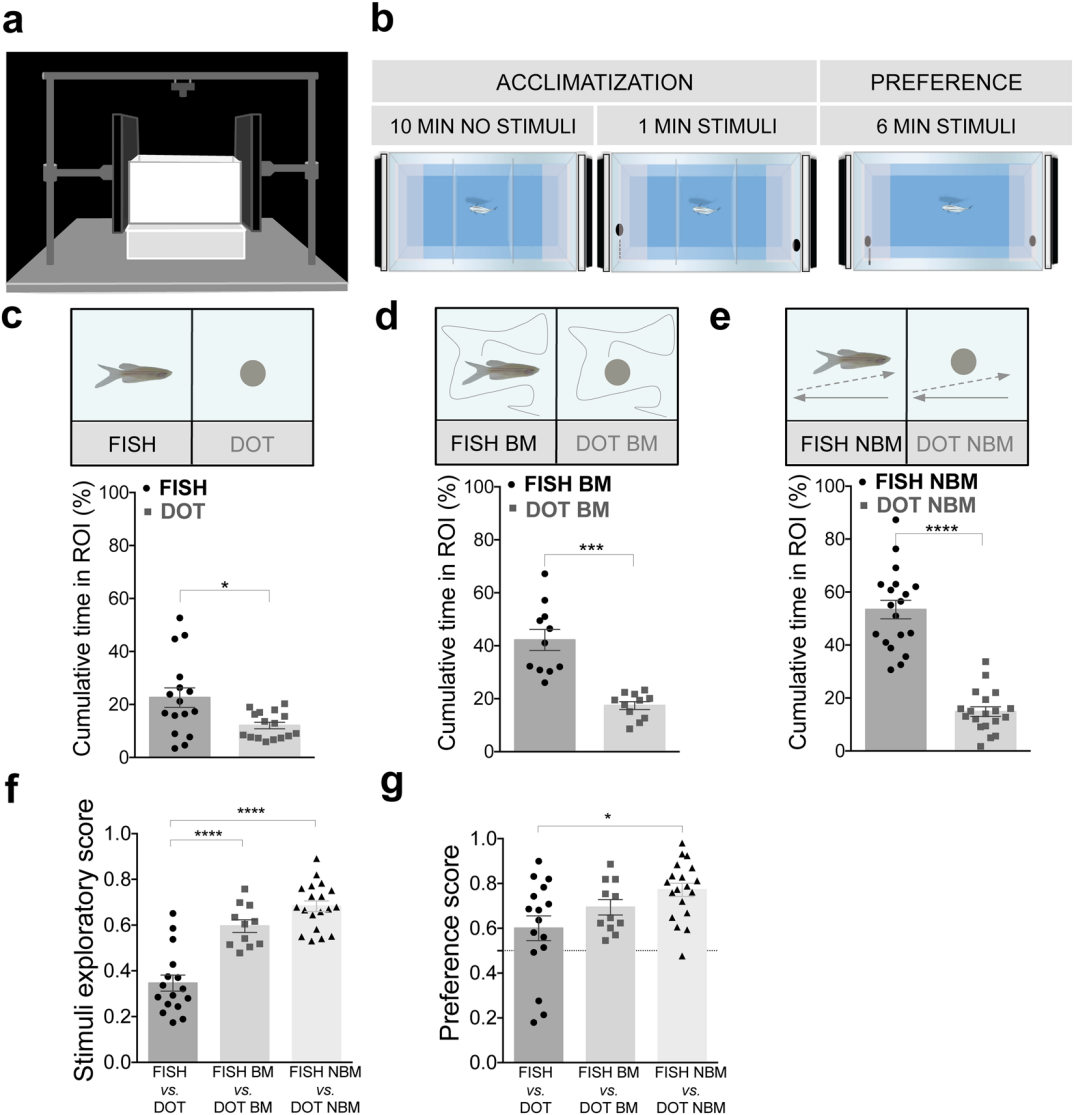
Conspecific form promotes zebrafish social affiliative behaviors. **(a)** Schematic of the behavioral setup. **(b)** Schematic of the experimental protocol: a focal fish is placed in the center of the experimental tank for an acclimatization phase, having visual access to two screens placed in each side of the tank, where an image of an empty tank is presented (background image). After 10 min, the stimulus appears in the screens for 1 min, then the partitions are lifted, and fish is allowed to explore the tank for 6 min (preference phase). The time spent in each side of the tank (ROI) is taken as a preference for the respective stimulus. **(c)** Zebrafish is allowed to choose between a static image of a fish vs. a static image of a dot. % Cumulative time fish spent near static fish image (FISH, black dots) vs. dot (DOT, grey squares, n = 16). **(d)** Zebrafish is allowed to choose between a fish vs. a dot moving with biological motion. % Cumulative time fish spent near fish with biological motion (FISH BM, black dots) vs. dot with biological motion (DOT BM, grey squares, n = 11). **(e)** Zebrafish is allowed to choose between a fish vs. a dot moving with non-biological motion. % Cumulative time fish spent near fish with non-biological motion (FISH NBM, black dots) vs. dot with non-biological motion (DOT NBM, grey squares, n = 19). **(f)** Motion increases the stimuli exploration. Comparison of the exploratory score between the experiments above described (stimuli exploratory score  =  time spent in both stimuli over total time). **(g)** Zebrafish preference towards conspecific form increases with motion. Comparison of the preference score (time spent in the conspecific form over time spent near both stimuli), between the experiments above described. Error bars indicate SEM. *P < 0.05, ***P < 0.001, ****P < 0.0001.

To explore visually mediated social attraction, we exposed zebrafish to two competing video playback stimuli that differed in two main visual features of conspecifics: conspecific form and BM. As a measure of preference, we compared the time a fish spent closer (within one-body length) to one of two competing stimuli (Fig. 1a,b). We first tested preference for conspecific form. Zebrafish were exposed to videos of static images of a fish vs. a dot with the same mean area and color as the fish images (Fig. 1c**)**. When given a choice between these two stimuli, zebrafish showed a significant preference for the conspecific form (comparison between % cumulative time fish spent in Fish vs. Dot: p = 0.04, n = 16, dz = 0.72, Fig. 1c and Supplementary Fig. S1d**)**. This finding suggested that fish discriminated the stimuli, and were attracted to the socially relevant stimulus. This preference towards conspecific form was enhanced when combined with BM (comparison between % cumulative time fish spent in Fish BM vs. Dot BM: p = 0.001, n = 11, dz = 1.89, Fig. 1d and Supplementary Fig. S1e) or non-biological motion (NBM) (Fish NBM vs. Dot NBM: p < 0.0001, n = 19, dz = 2.45, Fig. 1e and Supplementary Fig. S1f). Furthermore, zebrafish explored the stimuli significantly more when motion was present, exhibiting an exploratory score (as a measurement of engagement with any of the two stimuli: time spent near either stimuli over total time) higher for both BM (p < 0.0001, ds = 2.02; Fig. 1f) and NBM (p < 0.0001, ds = 2.78; Fig. 1f) when compared with static stimuli. The preference towards conspecific form, measured as a preference score (time spent near Fish stimulus over total time spent in either Fish or Dot stimuli), was slightly higher, but not significant, in the presence of BM (static (Fish vs. Dot) vs. BM (Fish BM vs. Dot BM): p = 0.28, ds = 0.50; Fig. 1g) and significantly higher in the presence of NBM (static (Fish vs. Dot) vs. NBM (Fish NBM vs. Dot NBM): p = 0.01, ds = 0.97; Fig. 1g). These results show that zebrafish discriminate conspecific form independently from motion; however, motion elicits a higher engagement and preference for the fish image.

**Figure 2:**
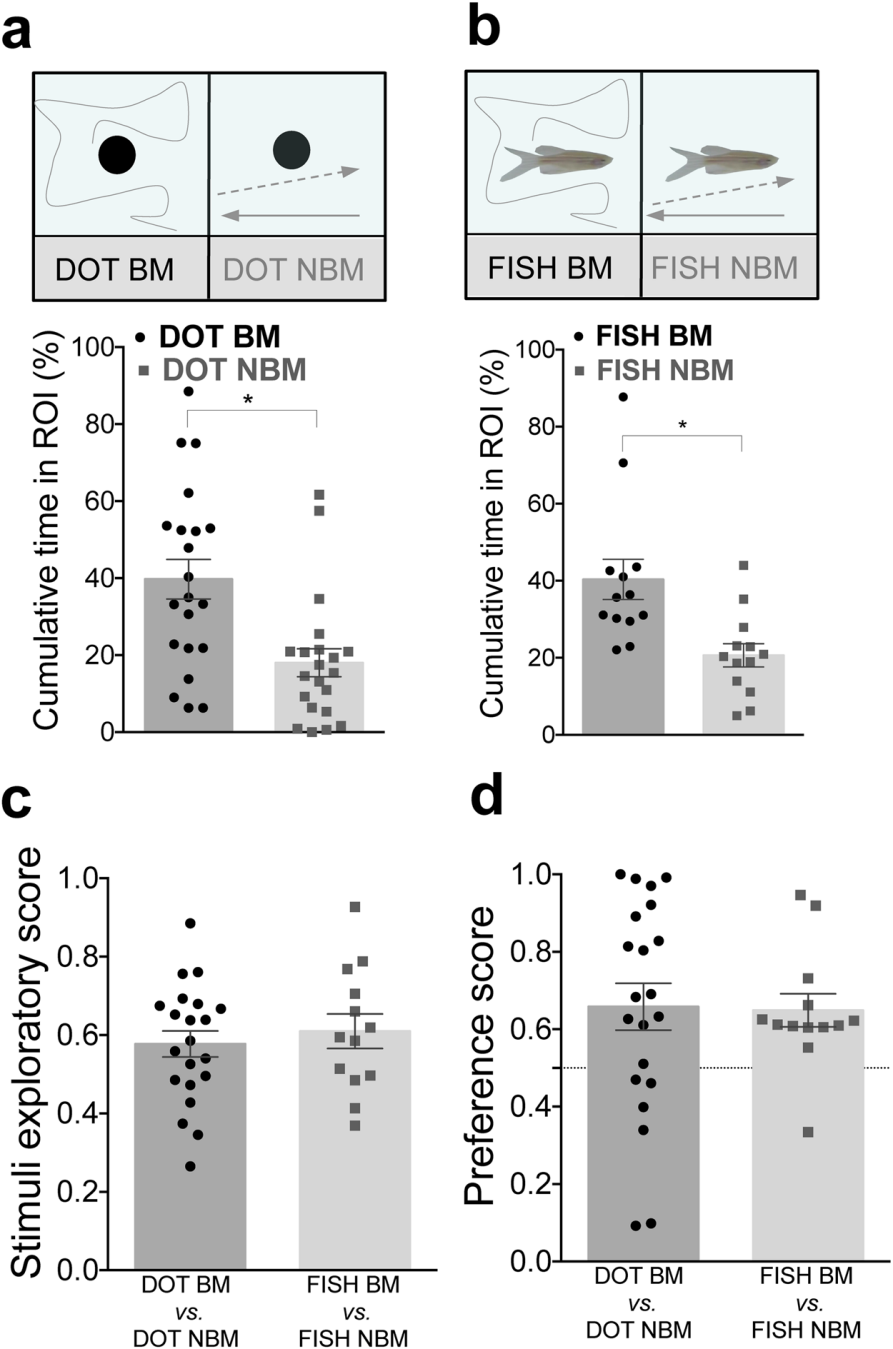
Biological motion promotes zebrafish social affiliative behaviors. **(a)** Zebrafish prefers to associate with a dot with biological motion (DOT BM) than non-biological motion (DOT NBM). % Cumulative time fish spent near DOT BM (black dots) vs. DOT NBM (grey squares, n = 21). **(b)** Zebrafish prefers to associate with a fish with biological motion (FISH BM) than a fish with non-biological motion (FISH NBM). % Cumulative time fish spent near FISH BM (black dots) vs. FISH NBM (grey squares, n = 13). **(c)** Comparison of the exploratory score between the experiments above described**. (d)** Comparison of the preference score towards biological motion between the experiments above described. Error bars indicate SEM. *P < 0.05.

Next, we tested zebrafish preference for BM. In the absence of conspecific form and color cues, fish showed a strong preference for BM (Fig. 2a**)**. When given a choice between a Dot animated with BM vs. a Dot with NBM, fish spent significantly more time closer to the Dot with BM (comparison of mean % cumulative time fish spent in Dot BM over Dot NBM: p = 0.02, n = 21, dz = 0.87, Fig. 2a and Supplementary Fig. S2a). This preference was maintained whether fish were exposed to videos of dots or conspecifics (Fish BM vs. Fish NBM: p = 0.01, n = 13, dz = 1.11 Fig. 2b and Supplementary Fig. S2b). No significant differences were observed in stimuli exploration (exploratory score: p = 0.56, ds = 0.21, Fig. 2c), or preference for BM (preference score: p = 0.48, ds = 0.04, Fig. 2d), suggesting that when the two competing stimuli have the same form and differ only in motion cues, form does not influence stimulus attraction or biological motion perception.

**Figure 3:**
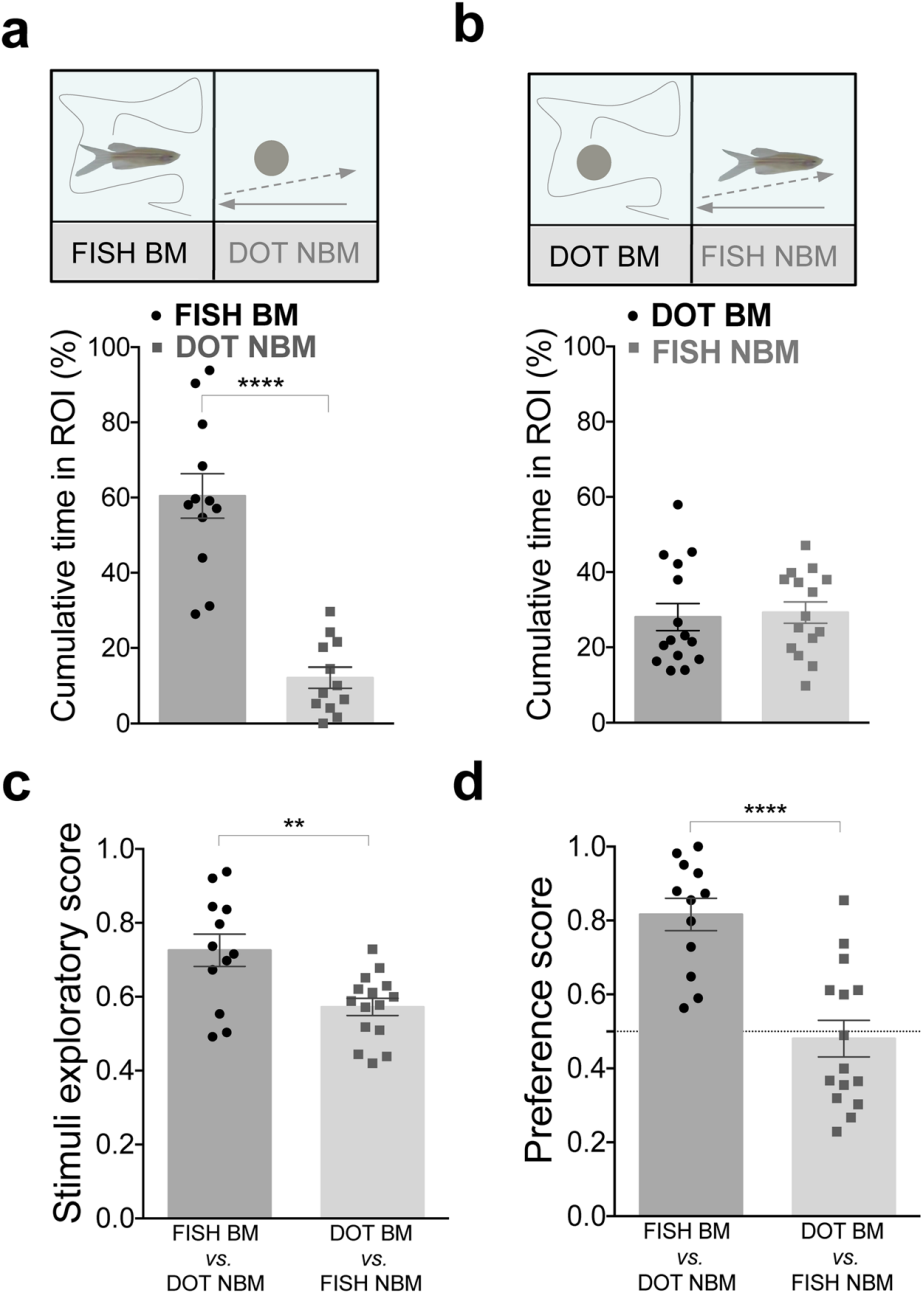
Congruent social stimuli promote social preference. **(a)** Zebrafish has a strong preference towards congruent social stimuli. % Cumulative time fish spent next to FISH BM (black dots) vs. DOT NBM (grey squares, n = 12). **(b)** Zebrafish does not exhibit preference for mismatched social stimuli. % Cumulative time fish spent next to DOT BM (black dots) vs. FISH NBM (grey squares, n = 15). **(c)** Zebrafish explores more the congruent than incongruent stimuli. Comparison of the stimuli exploratory score between congruent and incongruent stimuli. **(d)** Zebrafish prefers more the congruent than incongruent stimuli. Comparison of the preference score between social congruent and incongruent stimuli. Error bars indicate SEM. **P < 0.01, ****P < 0.0001.

Lastly, we tested preference for both biological shape and motion together. When given a choice between a Fish BM vs. Dot NBM, in which conspecific form was matched with biological motion and non-conspecific form with non-biological motion (i.e. both stimuli with congruent cues), zebrafish showed a strong preference for congruent social stimulus, spending significantly more time near the video of the conspecific than the dot (p < 0.0001, n = 12, dz = 2.31, Fig. 3a and Supplementary Fig. S3a). However, when exposed to mismatched stimuli (i.e. incongruent cues), a video of a Dot BM vs. a Fish NBM, zebrafish spent equivalent time near both stimuli (p = 0.77, n = 15, dz = 0.11, Fig. 3b and Supplementary Fig. S3b). Zebrafish explored more matched than mismatched stimuli (exploratory score: p = 0.003, ds = 1.26, Fig. 3c) and exhibited a significantly higher preference for the BM stimulus in the matched experiment (preference score: p < 0.0001, ds = 1.92, Fig. 3d). These results suggest that conspecific form and biological motion have to be matched to promote social attraction and preference.

Overall, these results indicate that zebrafish respond both to biological motion and to conspecific form and use this information either separately or in combination (congruent stimuli) to decide whether or not to interact with conspecifics.

### Elementary cues of biological motion elicit approach in zebrafish

Biologic motion can be decomposed into elementary animacy cues, such as changes in acceleration^35^ and start from rest, which indicates that there is an internal energy source that initiates the motion (aka self-propulsion)^36^. Newly hatched chicks and humans, although distant-related species, are both able to perceive these elementary cues of animacy when simple geometric forms are used (e.g. single dot). Such stimuli elicits affiliative behaviors in chicks, preferential attention in infant humans and perception of animacy by adult humans^35–37^.

Here, we aimed to investigate if zebrafish can also perceive these elementary animacy cues and whether they contribute to zebrafish affiliative behavior.

**Figure 4:**
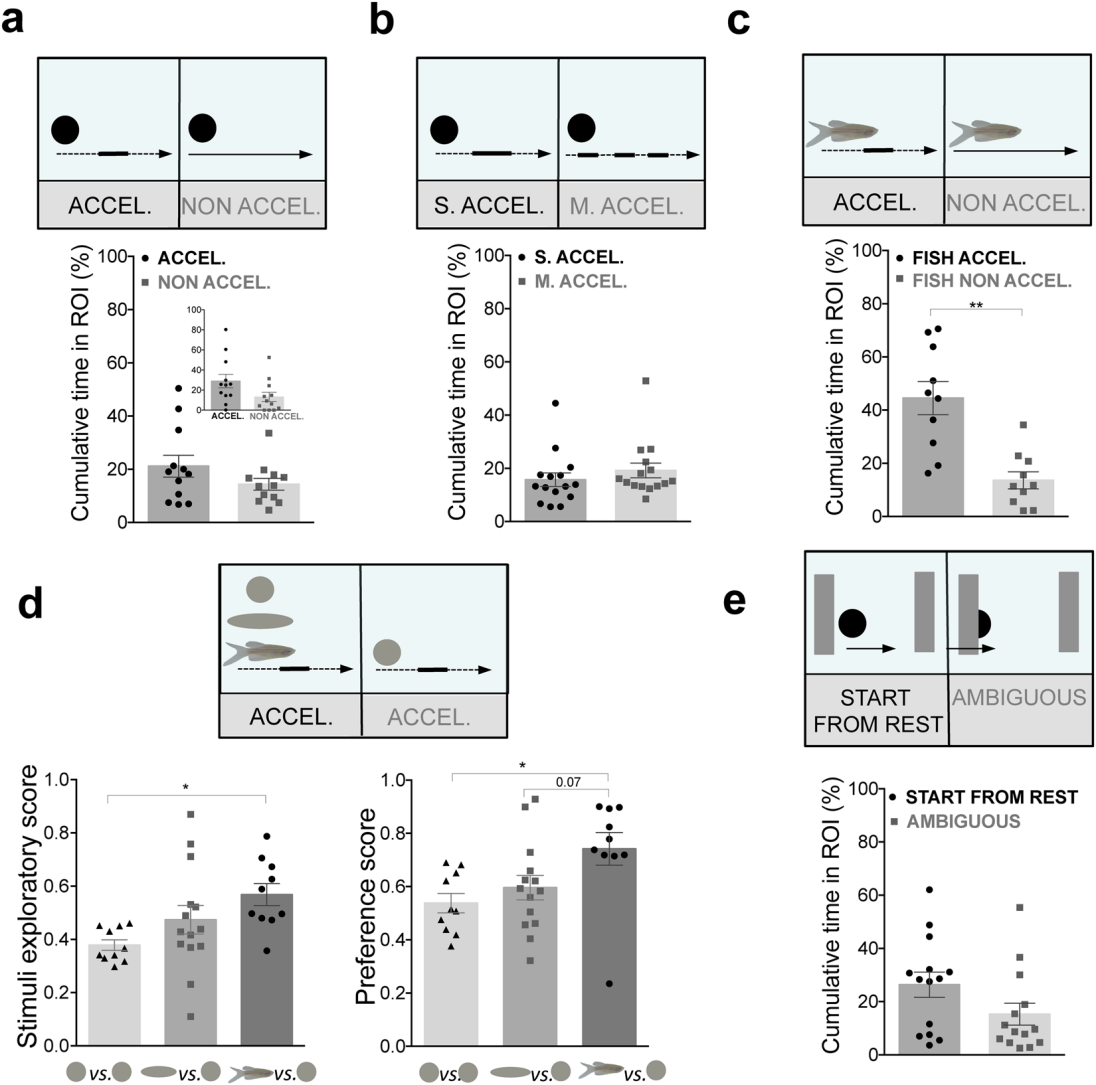
Zebrafish perceives elementary cues of biological motion and shape enhances this preference. **(a)** Zebrafish preference towards speed-changes (acceleration) cues. % Cumulative time fish spent next to a dot with speed-changes cues (ACCEL., black dot) vs. constant mean speed cue (NON ACCEL., grey squares) (n = 12). Depict of the % cumulative time in ROI during the first 2 min of the trial is shown. **(b)** Increasing the moments of acceleration do not increase preference towards speed-changes. % Cumulative time fish spent next to a single speed change (S.ACCEL., black dots) vs. multiple speed-changes (M. ACCEL., grey squares, n = 15). **(c)** Conspecific shape enhances attraction to speed-change cues. % Cumulative time fish spent next to a fish image with speed-changes (FISH ACCEL., black dots) vs. constant speed (FISH NON ACCEL., grey squares, n = 10). **(d)** Shape enhances attraction to acceleration cues. Changing from dot to elongated shape, and to conspecific form increases stimuli exploratory score and preference score. **(e)** Zebrafish preference towards start from rest cues. % Cumulative time fish spent next to a start from rest stimulus (START FROM REST, black dots) vs. an ambiguous stimulus (grey squares, n = 14). Error bars indicate SEM. *P < 0.05, **P < 0.01.

Following the work of Rosa-Salva *et al*.^35^ and Di Giorgio *et al*.^36^, we used a single black dot that always entered the screen from the left side and exited on the opposite side. In the first set of experiments, one of the screens presented a dot moving with speed changes along its trajectory (acceleration cue), while in the other, it moved with a constant mean speed (non-acceleration cue, Fig. 4a). When given a choice between the video with acceleration vs. non-acceleration cues, zebrafish spent more time, although not significant, closer to the acceleration cue during the 6 min trial (p = 0.38, n = 12, dz = 0.35, Fig. 4a), and with a medium-high effect size during the first 2 min of the test (p = 0.13, n = 12, dz = 0.70, Fig. 4a and Supplementary Fig. S4a). We then conducted experiments to test how to increase the response to acceleration. Increasing the moments of acceleration along its trajectory (multiple vs. single speed changes) did not significantly increase the % cumulative time near the acceleration stimulus (for the entire trial: p = 0.23, n = 15, dz = 0.27, Fig. 4b and Supplementary Fig. S4b). However, conspecific form enhanced attraction for the acceleration cue. When given a choice between a video of a fish image moving with speed changes vs. a fish image moving with a constant speed along its trajectory (Fig. 4c), zebrafish spent significantly more time near the fish with speed changes (for the entire trial: p = 0.008, n = 10, dz = 1.52, Fig. 4c and Supplementary Fig. S4c). Furthermore, zebrafish explored significantly more the stimuli during the entire trial when conspecific form was present in both screens (p = 0.0005, ds = 1.79, Supplementary Fig. S4d) along with a considerably higher, but not significant, preference towards speed-changes for the conspecific form over the dot (p = 0.08, ds = 0.80, Supplementary Fig. S4e**)**.

We also manipulated form (changing from a dot, to an elongated shape and finishing with an image of a fish) while maintaining the same speed changes along their trajectory (Fig. 4d and Supplementary Fig. S4f–n**)**. First, when exposed to both dots with speed-changes, zebrafish spent equally amount of time closer to each one, during the entire trial (p = 0.45, n = 10, dz = 0.32, Supplementary Fig. S4f–h). When given a choice between a dot vs. an elongated shape, both with speed-changes, zebrafish spent considerably, but not significantly, more time closer to the elongated shape (p = 0.07, n = 14, dz = 0.61, Supplementary Fig. S4i–k**)**. Finally, when exposed to an image of fish vs. a dot, both with speed-changes, zebrafish spent significantly more time closer to the stimulus with socially relevant cues (p = 0.04, n = 10, dz = 1.44 Supplementary Fig. S4l–n**)**. Zebrafish explored more the stimuli from the dot to elongated object to a fish form (for the entire trial, comparison between Dot vs. Dot and Dot vs. Elongated: p = 0.26, ds = 0.60; Dot vs. Elongated and Dot vs. Fish: p = 0.26, ds = 0.54; Dot vs. Dot and Dot vs. Fish: p = 0.03, ds = 1.85, one-way ANOVA with Holm-Sidak´s multiple comparisons test, Fig. 4d) and increased their preference with conspecific form (for the entire trial, comparison between Dot vs. Dot and Dot vs. Elongated: p > 0.99, ds = 0.39; Dot vs. Elongated and Dot vs. Fish: p = 0.07, ds = 0.80; Dot vs. Dot and Dot vs. Fish: p = 0.01, ds = 1.28, Kruskal-Wallis with Dunn´s multiple comparisons, Fig. 4d).

Another characteristic that distinguishes animate from inanimate objects is the ability to initiate motion (i.e. start from rest), which is also a cue of self-propulsion. To test if zebrafish perceived this self-propulsion cue, two different stimuli were presented, adapted from Di Giorgio *et al*.^36^: a) video with a dot that initiated its motion by itself and left the screen (start from rest stimulus), and b) a video of a dot that appeared in the screen already in motion and then stopped (ambiguous stimulus) (Fig. 4e and Supplementary Fig. S4o,p). When given a choice between these two stimuli, zebrafish spent more time, but not significantly, closer to the start from rest stimulus than ambiguous stimulus during the entire 6 min trial (p = 0.13, dz = 0.56, n = 14, Fig. 4e and Supplementary Fig. S4o,p) and during the first 2 min trial (p = 0.20 dz = 0.52, n = 14, Fig. S4p).

Overall, these results indicate a moderate zebrafish preference towards elementary cues of BM, speed-changes and the onset of motion, which increases with conspecific form.

### Oxytocin signaling is differentially involved in the perception of biological motion and conspecific form

**Figure 5:**
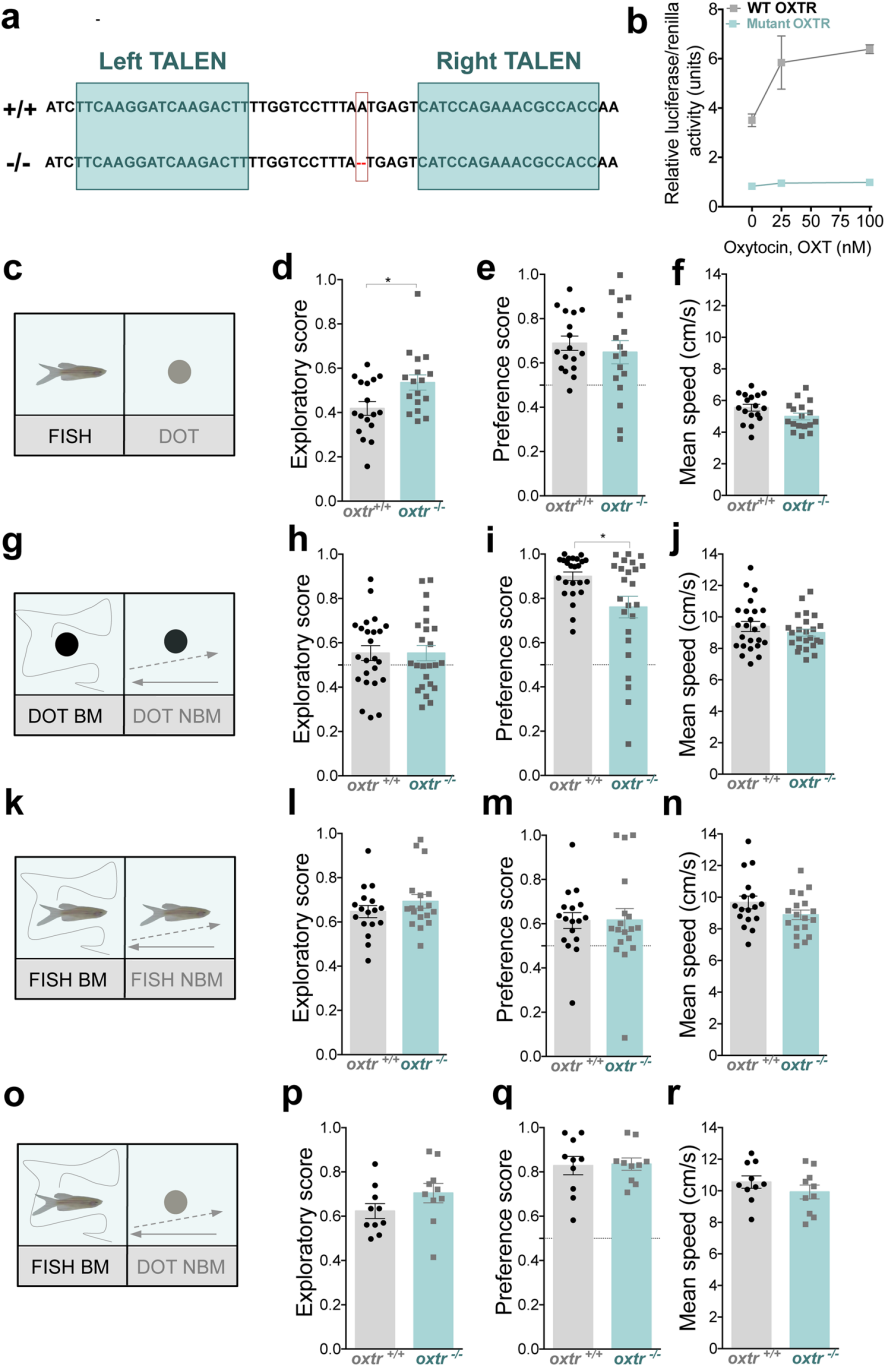
Oxytocin signaling is differently involved in the perception of biological motion and conspecific form. (**a)** Generation of germline transmitting *oxtr* mutant. TALEN sites targeting the 1^st^ exon of the *oxtr* gene. Single bp mutation generates a truncated receptor. **(b)** Different concentrations of synthetic isotocin, also known as oxytocin in fish literature, increased cAMP reporter activity of the WT-transfected OXTR, but not the mutant. **(c)** WT (*oxtr*^(+/+)^) and *oxtr* mutant (*oxtr*^(−/−)^) fish are allowed to choose between a static fish image (FISH) vs. a static dot (DOT). **(d)** Comparison of exploratory score between *oxtr*^(+/+)^ (grey bars, n = 17) and *oxtr*^(−/−)^ (cyan bars, n = 16). **(e)** Comparison of preference score towards social cues between *oxtr*^(+/+)^ (grey bars, n = 17) and *oxtr*^(−/−)^ (cyan bars, n = 16). **(f)** Comparison of mean speed between *oxtr*^(+/+)^ (grey bars, n = 17) and *oxtr*^(−/−)^ (cyan bars, n = 16). **(g)** Fish *oxtr*^(+/+*)*^ and *oxtr*^(−/−)^ are allowed to choose between a dot moving with biological motion (DOT BM) vs. a non-biological motion (DOT NBM). **(h)** Comparison of exploratory score between *oxtr*^(+/+)^ (grey bars, n = 24) and *oxtr*^(−/−)^ (cyan bars, n = 24). **(i)** Comparison of preference score towards social cues between *oxtr*^(+/+)^ (grey bars, n = 24) and *oxtr*^(−/−)^ (cyan bars, n = 24). **(j)** Comparison of mean speed between *oxtr*^(+/+)^ (grey bars, n = 24) and *oxtr*^(−/−)^ (cyan bars, n = 24). **(k)** Fish *oxtr*^(+/+)^ and *oxtr*^(−/−)^ are allowed to choose between a fish moving with biological motion (FISH BM) vs. a fish image moving with non-biological motion (FISH NBM). **(l)** Comparison of exploratory score between *oxtr*^(+/+)^ (grey bars, n = 17) and *oxtr*^(−/−)^ (cyan bars, n = 18). **(m)** Comparison of preference score towards social cues between *oxtr*^(+/+)^ (grey bars, n = 17) and *oxtr*^(−/−)^ (cyan bars, n = 18). **(n)** Comparison of mean speed between *oxtr*^(+/+)^ (grey bars, n = 17) and *oxtr*^(−/−)^ (cyan bars, n = 18). **(o)** Fish *oxtr*^(+/+)^ and *oxtr*^(−/−)^ are allowed to choose between a fish moving with biological motion (FISH BM) vs. a dot with non-biological motion (DOT NBM). **(p)** Comparison of exploratory score between *oxtr*^(+/+)^ (grey bars, n = 10) and *oxtr*^(−/−)^ (cyan bars, n = 10). **(q)** Comparison of preference score towards social cues between *oxtr*^(+/+)^ (grey bars, n = 10) and *oxtr*^(−/−)^ (cyan bars, n = 10). **(r)** Comparison of mean speed between *oxtr*^(+/+)^ (grey bars, n = 10) and *oxtr*^(−/−)^ (cyan bars, n = 10). Error bars indicate SEM. *P < 0.05.

To determine if oxytocin modulates the ability of zebrafish to perceive biological motion and conspecific form, we used the transcription activator-like effector nucleases (TALEN) genome editing method to generate an oxytocin receptor *oxtr* mutant line, in which a single nucleotide deletion caused a frame shift mutation leading to premature stop codon and a truncated OXTR protein **(**Fig. 5a**)**. To confirm that the OXTR was not functional, we have compared ligand-induced signaling of wild type (WT) and mutated forms of zebrafish *oxtr*, bearing the same single nucleotide indel. To assess OXTR signaling, we co-transfected HEK293 cells with a luciferase reporter, which is driven by the cAMP response element (CRE) together with either WT or mutant *oxtr* forms. HEK293 cells, which were transfected with WT OXTR but not with its mutant cDNA form displayed a dose-dependent luciferase activity in response to increasing concentrations of recombinant oxytocin indicating that the mutation we have generated lead to an inactive OXTR **(**Fig. 5b**)**.

We then compared wild type (WT) *oxtr*^(+/+)^ with *oxtr*^(−/−)^ mutant fish for their preference towards conspecific form by exposing both genotypes to static images of Fish vs. Dot **(**Fig. 5c**)**. Compared to WT fish, *oxtr*^(−/−)^ mutant fish spent significantly more time exploring both stimuli (comparison of stimuli exploratory score between *oxtr*^(+/+)^, n = 17, and *oxtr*^(−/−)^, n = 17: p = 0.01, ds = 0.89, Fig. 5d and Supplementary Fig. S5a,b) but no differences were observed between WT and mutant fish in their preference towards an image of the Fish over a Dot (comparison of preference score: p = 0.51, ds = 0.23, Fig. 5e and Supplementary Fig. S5a,c).

We next compared the two different genotypes for their preference towards biological motion (Fig. 5g). When given a choice between videos of a Dot BM vs. Dot NBM, both genotypes equally explored the stimuli (exploratory score between *oxtr*^(+/+)^, n = 24, and *oxtr*^(−/−)^, n = 24: p = 0.99, ds =  0.001, Fig. 5h and Supplementary Fig. S5d,e); however mutant *oxtr*^(−/−)^ fish showed a significantly lower preference for the Dot BM (comparison of preference score: p = 0.02, ds = 0.68, Fig. 5i and Supplementary Fig. S5d,f). This difference between the two genotypes towards BM preference was not observed if instead of dots we used conspecific form: Fish BM *vs*. Fish NBM (Fig. 5k). No significant differences were observed between WT and *oxtr*^(−/−)^ fish for stimuli exploratory score (p = 0.27, ds = 0.38, Fig. 5l and Supplementary Fig. S5g,h) and preference score towards Fish BM (p = 0.56, ds = 0.01, Fig. 5m and Supplementary Fig. S5g,i).

Finally, we compared WT *oxtr*^(+/+)^ and *oxtr*^(−/−)^ mutant fish for their preference towards conspecific form and motion when presented together and matched, Fish BM vs. a Dot NBM (Fig. 5o). We observed that both genotypes strongly engaged with the social stimulus. *Oxtr*^(−/−)^ mutants exhibited a slightly, but not significant, increase in the stimuli exploratory score (p = 0.16, n = 10 for both genotypes, ds = 0.65, Fig. 5p and Supplementary Fig. S5j,k) and no significant differences were observed between the two genotypes in the preference score towards social stimulus (p = 0.89, n = 10 for both genotypes, ds = 0.06, Fig. 5q and Supplementary Fig. S5j,l). In all experiments performed, no differences were observed in mean swimming speed between *oxtr*^(+/+)^ vs. *oxtr*^(−/−)^ mutant fish, indicating no motor impairment in the *oxtr*^(−/−)^ mutant (for mean speed in static Fish vs. static DOT, p = 0.07, ds = 0.63, Fig. 5f; Dot BM vs. DOT NBM, p = 0.32, ds = 0.29, Fig. 5j; Fish BM vs. Fish NBM, p = 0.13, ds = 0.53, Fig. 5n; Fish BM vs. DOT NBM, p = 0.30, ds = 0.48, Fig. 5r).

We also compared WT and *oxtr*^(−/−)^ for their ability to perceive elementary cues of biological motion. When given a choice between a video with acceleration vs. non-acceleration cues (Supplementary Fig. S5m) no differences were observed between WT and *oxtr*^(−/−)^ mutant fish in stimuli exploratory score (p = 0.56, ds = 0.18; n = 21 *oxtr*^(+/+)^, n = 25 *oxtr*^(−/−)^ Supplementary Fig. S5n), preference score (p = 0.86, ds = 0.13, n = 21 *oxtr*^(+/+)^, n = 25 *oxtr*^(−/−)^, Supplementary Fig. S5o) and mean speed (p = 0.50, ds = 0.20, n = 21 *oxtr*^(+/+)^, n = 25 *oxtr*^(−/−)^, Supplementary Fig. S5p).

When given a choice between start from rest or ambiguous stimulus **(**Supplementary Fig. S5q**)**, mutant *oxtr*^(−/−)^ fish explored significantly more the stimuli than the WT (p = 0.004, ds = 0.99, n = 22 oxtr^(+/+)^, n = 21 *oxtr*^(−/−)^, Supplementary Fig. S5r) but no differences were observed in preference score (p = 0.13, ds = 0.47, n = 22 oxtr^(+/+)^, n = 21 *oxtr*^(−/−)^, Supplementary Fig. S5s) and mean speed (p = 0.63, ds = 0.25, n = 22 oxtr^(+/+)^, n = 21 *oxtr*^(−/−)^, Supplementary Fig. S5t) between the two genotypes.

These results suggest that oxytocin is implicated in the overall perception of biological motion, but not in the perception of individual basic elementary animacy cue that compose the biological motion.

Altogether, our results suggest that visual cues such as conspecific form and BM, either alone or matched together, are important to induce affiliative behaviors, with BM, *per se* or together with conspecific form, promoting a higher engagement and preference of the fish. Furthermore, the perception of BM, but not biological shape, is affected by partial disruption of OXT signaling.

## Discussion

Our results add insights into the perceptual mechanisms of social affiliative behaviors in zebrafish. We demonstrated that zebrafish integrates two main visual cues, conspecific form and BM, that act synergistically to inform the decision about whether or not to interact with conspecifics. Separately, each of these cues also promotes affiliative behaviors by itself. Conspecific form, *per se*, is sufficient for fish to discriminate between social and non-social stimuli. However, BM triggers a higher attraction to the stimuli and a faster discrimination between the two. Furthermore, OXT signaling modulates the perception of biological motion, but not biological shape. Thus, our results support that oxytocin signaling participates in basic perceptual mechanisms of social affiliation shared across species and crucial for the maintenance of sociality.

In our study, zebrafish used distinct features of conspecific form and BM to perceive social information and make social decisions. Conspecific form, alone, was sufficient for zebrafish to discriminate between social and non-social stimuli, and was most relevant in the last minutes of the trial. However, this effect was stronger and faster if motion cues were present.

BM, alone, assessed by using a single dot moving with the same motion of a zebrafish but lacking conspecific form information, promoted a higher engagement with the stimuli than form alone, and allowed a rapid discrimination (i.e. this effect was more robust during the first minutes of the trial) between social and non-social cues. These different dynamic responses for conspecific form and BM may be due to the fact that biological motion causes rapid changes in the environment, and so, fish needs to quickly gather as much information as possible. BM has been shown, in many species, as a key feature to identify the presence of conspecifics, to infer their actions and, overall, to guide social behaviors. Humans have the ability to identify moving individuals from point-light displays (i.e. lights placed on the joints of subjects walking in a dark background) and to recognize their actions, emotional states and gender^38–40^. Similarly, several other species, such as chimpanzees, cats, newly-hatched chicks, and medaka fish, among others, also perceive biological motion from point-lights simulating their motion, suggesting a broadly underlying conserved mechanism among vertebrates, that is probably innate^41–44^. However, using this point-light display approach, the studies are not only including information about motion, but also form (or contour) from the global configuration of all the points, even though no explicit form (appearance) information is provided. To disentangle form from motion, Shibai and collegues^45^ have showed that the biological motion of medaka can be decomposed into body-shape motion and trajectory-motion, and each component alone can attract its attention^45^.

In our work, conspecific form was not required to establish a preference for BM nor did it affect the interest in BM when the same form was presented in both stimuli. Interestingly, we observed that not only BM but also NBM strengthened the effect of conspecific form. The reason for this result is not known, but perhaps, when conspecific form is presented with NBM, fish detects an incongruence in the social form that might be more complex to resolve and demands more attention. When an incongruent social stimulus (Fish NBM) was presented together with a congruent one (Fish BM, Fig. 2b), zebrafish exhibited a clear preference for the latter one (Fish BM), indicating that zebrafish can bind the features together, as demonstrated previously^46^. If both videos presented mismatched features (incongruent stimuli), as observed when given a choice between Fish NBM vs. Dot BM, fish failed to bind the features and did not show a stimulus preference (Fig. 3b).

Furthermore, we decomposed BM into elementary animacy cues and showed for the first time, that zebrafish can perceive and is attracted to speed-changes. This ability to perceive basic animacy cues has been demonstrated in few other species so far (e.g. chicks and humans)^35,36^. In newly-hatched chicks, studies have shown that acceleration (speed changes cues) elicits predispositions for social affiliation^35^, activating brain regions that also respond to the sight of conspecifics^47^. Our results using a phylogenetically distantly-related species support that this perceptual mechanism may be broadly conserved across vertebrates.

Our experiments were not designed to assess the absolute contribution of conspecific form and biological motion cues, since we measured preferences for competing stimuli. However, we observed that the BM highly increased the preference towards conspecific form and the combination of the two elicited a robust discrimination between social and non-social stimuli, suggesting that these two features may act synergistically. Moreover, even though our study was intended to assess the role of visual cues only, we recognize that, in nature, other sensory modalities (e.g. olfaction, auditory and lateral line mechanoreception) are present and that social perception is most probably multimodal, integrating cues in these different sensory modalities.

Based on our results, we hypothesize that the process for extracting socially relevant visual information may follow a two-step mechanism. First, fish rapidly attend to BM and subsequently use information on conspecific form to sustain their social preference. Observations in other species support this hypothesis. Studies in monkeys and clinical studies in humans have shown that BM and form are processed in separate areas of the visual cortex^48,49^. In teleost fish, the visual processing center is the optic tectum, where the superficial layers receive retinal afferents and process visual input^50^. As found in other species, where sensory brain areas express oxytocin receptor^26,27^ also in fish both olfactory and visual brain areas seem to be enriched with OXTR^28^. With receptors expressed in such key sensory areas of the brain, OXT could rapidly modulate the extraction of social information, and this information would then be processed in higher-order brain regions that are activated during social interactions and that also express OXTR^18,24^. In support of this hypothesis, previous studies on dogs and humans have demonstrated that OXT increases the perception of biological motion using point-light displays^30,31^. Additionally, our study, using a genetic approach to partially disrupt OXT signaling, showed for the first time in zebrafish, that OXT modulates visual perception of biological motion, but not biological shape. In the absence of conspecific form information, fish lacking a functional OXTR showed a decreased preference for BM compared to WT. This effect cannot be explained by impaired locomotion or stimulus exploration, since those did not differ between *oxtr*^(−/−)^ mutant and WT fish. This effect was not observed in more elementary cues of biological motion, such as speed-changes.

The lack of OXT signaling also influenced stimuli exploration, especially when stimuli contained conspecific form information, suggesting that mutant fish might have more difficulty extracting information from the stimuli and therefore spend more time near both presented stimuli. Previous studies^51^ support this idea by showing that, in rodents, *oxtr* deletion in the anterior olfactory cortex causes increased exploration of conspecifics, possibly because these mice were less efficient in extracting social information because oxytocin no longer amplifies odor representations^51^.

When both BM and conspecific form features are presented together, the conspecific form compensates the decreased perception of biological motion, and no differences are observed between WT and mutant fish. The reason for this is yet only speculative, but perhaps OXT is acting on receptors differentially expressed in the visual processing areas. Of note, in our work, we only disrupted the function of one of two OXTR subtypes. While mammals have only one OXTR type, teleost fish have two due to gene duplication^52^, and at the moment, we have no information on how the two receptor types are distributed in the zebrafish brain. Thus, so far, we can not exclude a role of the other OXT receptor subtype in conspecific form and BM perception. Furthermore, the reduced effect observed in BM perception by the *oxtr*^(−/−)^ mutant fish suggests that OXT is not acting alone and other neuromodulators/neurotransmitters should be explored. We also cannot rule out the hypothesis that the lack of effects observed for social shape can be also due to the cross-talk between oxytocin and arginine-vasotocin receptors^53^.

Overall, our work adds insights into the perception of socially relevant visual stimuli and the contribution of OXT in a species that, though phylogenetically distant from humans, also uses primary visual information to guide social interactions with conspecifics. Research on conspecifics interactions is of particular translational interest since sensory processes serving social interactions have been shown to be compromised in autism spectrum disorders. Children with autism are less efficient in discriminating biological from non-biological motion cues^33^. Similarly, infants with autism do not show a spontaneous preference for biological point light animations^54^. Furthermore, deficits in the oxytocin system have been implicated in several atypical social behaviors across species. Thus our work supports the role of OXT signaling in fundamental and shared circuitry that is probably critical for social information extraction and processing.

## Methods

### Fish and housing conditions

Adult males, zebrafish, *Danio rerio* [WT and oxytocin receptor mutant (*oxtr* ^-/-^) lines from a mixed TL background] were kept in mixed sex groups (10 adults/L) in a recirculation life support system (Tecniplast) with the following parameters: 28 °C, pH 7.0, conductivity 1000 *µ*S/cm, 14 L:10D light:dark cycle. Fishes were fed with a combination of live food (*Paramecium caudatum* and *Artemia salina)* and commercial processed dry food (Gemma). Husbandry protocols, water chemistry and health program have been described previously^55^. Since 2016, the colony is free of all known pathogens, in particular, pre-filter sentinels tested negative for Mycobacteria and *Pseudoloma neurophilia*. All experiments were conducted in accordance with standard operating procedures of the Instituto Gulbenkian de Ciência Ethics Committee and DGAV-Direcção Geral de Alimentação e Veterinária, Portugal, with the permit number 0421/000/000/2015.

### Generation of germline transmitting *oxtr* mutant

The *oxtr* mutant line (ZFIN ID: ZDB-ALT-190830-1) was generated using the transcription activator-like effector nuclease (TALEN) genome-editing tool **(**Fig. 5a**)**. TALEN-based *oxtr* mutant fish was generated as described elsewhere^56^.

Briefly, plasmid constructs, TAL3330 and TAL3331, encoding nuclease-fused TALE domains targeting *oxtr* gene were generated by the NIH-funded Resource to Target Zebrafish Genes with Engineered Nucleases, and obtained from the Addgene plasmid repository (Addgene plasmids # 42793 and # 42794; http://n2t.net/addgene:42793 and http://n2t.net/addgene:42794; RRID:Addgene_42793 and RRID:Addgene_42794, respectively). The plasmids were linearized using PmeI enzyme [New England Biolabs (NEB)], and RNA was synthesized from 1 μg of linearized plasmids using mMachine mMessage T7 ultra kit (Ambion) and purified using Lithium chloride precipitation technique. The purified RNA was pooled and 2 nl of 300 ng/μl RNA were injected into 1-cell stage embryo. Hotshot technique was utilized for genomic DNA preparation^57^. Briefly, 1-day-old embryos or fin-clips of adult fish were placed in PCR tubes, with 50 μl of lysis buffer (50 mM NaOH) and incubated at 95 °C for 30 min. The samples were then neutralized by the addition of 5 μl of 1 M Tris–HCl (pH 7.5) and 2 μl were taken for 25 μl of PCR mix. Initial screening of embryos was performed using restriction fragment length polymorphism analysis (RFLP) of pcr products using *MseI* enzyme [New England Biolabs (NEB)]. Germline transmitting *oxtr*^(−/−)^ was identified and propagated by outcrossing with WT fish. We verified by RT-PCR and sequencing that the mutant mRNA did not undergo non-sense mediate RNA decay, which is considered as a major factor in eliciting gene compensation^58^. For subsequent experiments, sanger sequencing was performed on PCR products to identify *oxtr*^(−/−)^ mutants.

### Plasmids

Full-length *oxtr* (NM_001199370) was amplified by PCR from mRNA isolated from embryos at 72 hours post fertilization and cloned into pCS2+ expression vector using EcoRI and XbaI restrictions sites. A mutant form of the receptor *mut* oxtr (ΔA41) was generated by PCR-based site-directed mutagenesis of *oxtr* cDNA. The mutated cDNA fragment was subsequently subcloned into the pCS2^+^ plasmid containing WT *oxtr* using EcoRI and EcoNI restriction sites and confirmed by nucleotide sequencing. Oligonucleotide primers that were used to amplify DNA templates for *oxtr* and *mut oxtr* amplification reactions are described in Supplementary Table 1.

### Transcriptional activation assay

Transcriptional activation assay was performed in HEK-293T cells using pCREB – Luc vector (Clontech) together with the indicated pCS2-based expression vectors^59^. HEK-293T cells were grown in 48-well plates and transfected (at 60% confluence) with a total amount of 1.0 μg/well of DNA using standard calcium phosphate transfection method. Renilla expression vector (Promega) was applied for the normalization of the transfections efficiency. In some experiments, 24 hours post transfection semi-confluent cells were grown overnight in the serum free DMEM (Sigma-Aldrich) and then incubated in the presence of different concentrations of synthetic isotocin peptide (Bachem, H-2520-0001, Germany), A.K.A oxytocin in zebrafish, or forskolin (Sigma-Aldrich, F3917). After 2.5 hours stimulation proteins were harvested in 100 μl of cell culture lysis reagent (Promega) and the crude protein extracts were subjected to the measurements of cAMP or renilla reporter activity using Turner BioSystems reader (Promega). Four experiments were performed, each repeated three times. The results are presented as relative luciferase/renilla (arbitrary units) ± SEM (Fig. 5b**)**.

### Fish genotyping

For individual genotyping genomic DNA was extracted from adult fin clips using the procedure described by Blechman *et al*.^59^. The region of interest was amplified by PCR and sequenced using the following primers: sense 5′ TGCGCGAGGAAAACTAGTT-3′, antisense 5′ AGCAGACACTCAGAATGGTCA-3′.

### Video-playbacks

#### Behavioral setup

The test tank measured 29.5 ×14.5 ×11 cm. Individual focal zebrafish were placed in a central compartment restricted by two removable transparent partitions during the acclimatization period. Two LCD monitors (Asus, LCD monitor VG248, Full HD1080, 144 Hz rapid refresh rate) were positioned on opposite sides of the tank **(**Fig. 1a**)**. The walls of the tank, except those adjacent to the monitors, were opaque (white) and the whole apparatus was covered in black material to prevent the influence of external visual stimuli. A third screen, connected to the same computer, was used to control and synchronize the two LCD screens (TightVNC remote control software). The test tank was illuminated with infrared LEDs, as described above.

#### Experimental procedure

Fish were isolated overnight in opaque tanks (10.5 ×10.5 ×10.5 cm) at 28 °C and 14 L:10D photoperiod. The next day, individual fish were placed in the central compartment of the experimental tank for an acclimation phase **(**Fig. 1b**)** during which both LCD screens displayed a video of a tank containing water (Empty tank). After 10 min, each monitor displayed a specific video stimulus. After 1 min, the partitions were lifted and the fish was allowed to explore the arena for 6 min while video-recorded for offline behavioral tracking. The presentation of the stimuli was balanced between the two screens.

Each fish was only tested once to avoid habituation to the behavioral setup.

We validated our video playback system by comparing the shoal preference response of zebrafish to videos of a shoal vs. real conspecifics. In both cases, zebrafish spent significantly more time near the compartment containing the shoal vs. an empty compartment (for a real shoal vs. empty compartment in a shoal preference setup with a unidirectional mirror: p < 0.0001, n = 17, Supplementary Fig. S1a; for a video of a shoal vs. a video of an empty tank in a videoplayback system: p < 0.0001, n = 14, Supplementary Fig. S1b). No differences were obtained when social preference score was compared between the two experiments (p = 0.82, Supplementary Fig. S1c**)**. Thus, our video playback of social stimuli elicits a similar behavioral response to that of a real fish.

#### Stimuli

The stimuli used in video playbacks were recorded with a goPro camera (goPro hero3+, 60 fps, 1080 pixel resolution) placed in front of a tank with the remaining walls opaque to exclude external influences.

All manipulations of the stimuli were done using Adobe AfterEffects software (CC 2015 Adobe Systems Inc, San Francisco, CA, USA), unless noted otherwise. The stimuli were displayed on the screens as real size images.

We chose to present only one single element in our stimuli, even though our optimization had shown a more robust affiliative response to a shoal of fish. Our rational is that interactions between fish and shoal are more difficult to parameterize.

The following stimuli were used in the experiments:

*Empty tank*– Image of a water-filled empty tank. This image was used as a background for the acclimatization phase (shown in Supplementary Videos 1 and 2**)**.

*Fish BM video* - A fish was transferred to the tank and recorded after 1 h of acclimatization. We used 8-seconds of recording in which the fish was closest to the screen (to avoid major changes in stimulus size and to trigger affiliative behaviors) (shown in Supplementary Video 1**)**.

*Dot with Biological Motion (Dot BM video)* – Custom-tracking software was used to extract the fish movement and centroid coordinates, and to replace the fish by a dot, so that the dot moved with the fish’s original movement (Python Video Annotator, https://pythonvideoannotator.readthedocs.io/en/master/index.html). The area and color of the dot were matched to the mean area and color of real zebrafish measured from several video snapshots using FIJI software (Schindelin, J. *et al*., 2012) (shown in Supplementary Video 1**)**.

*Dot with Non-Biological Motion (Dot NBM) –* A dot was made to move with different linear trajectories but the same mean speed as the dot in the Dot BM video stimulus. The dot entered from one side of the screen and moved linearly to exit the opposite side. The image re-entered the screen from the side it exited (changes in direction were not performed on screen, since they are a cue for biological motion) (shown in Supplementary Video 1**)**.

*Fish with Non-biological Motion (Fish NBM video)* – Same as Dot NBM, but dot was replaced by fish images (shown in Supplementary Video 1**)**.

*Speed changes video* – We used a method adapted from^35^. The dot always entered from the left side of the screen and exited the opposite side. During its motion, the dot accelerated for about one third of its trajectory and decelerated back to its initial speed around two thirds of its trajectory (shown in Supplementary Video 2**)**.

*Constant-speed video* - The dot always entered from the left side of the screen and exited on the opposite side, moving with a constant speed (shown in Supplementary Video 2**)**.

*Multiple Speed-changes*– Same as Speed changes video, but with multiple moments of speed changes (shown in Supplementary Video 2**)**.

*Fish Speed changes video* – Same as Speed-changes video but dot was replaced by an image of a fish (shown in Supplementary Video 2**)**.

*Fish Constant-speed video* - Same as Constant-speed video but dot was replaced by an image of a fish (shown in Supplementary Video 2**)**.

*Speed-changes with changing shape* – Same parameters as in the speed-changes video but the dot was replaced by either an elongated shape with the same mean color and mean area of the fish images, or an image of a fish (shown in Supplementary Video 2**)**.

*Start from rest Motion video* – We used a method adapted from^36^. A grey bar was placed at the beginning and end of the tank. A stationary dot was present in the screen, at the edge of the first bar. After 2 sec, it moved across the screen, disappearing behind the second bar (shown in Supplementary Video 2**)**.

*Ambiguous video* – This stimulus was also adapted from Di Giorgio *et al*.^36^. The dot entered the screen from behind the first bar, stopped near the second bar, and stayed immobile on the screen for 2 sec. The dot was visible on screen for the same amount of time as in start from rest motion videos (shown in Supplementary Video 2**)**.

### Behavioral analysis

In all behavioral tests, zebrafish were recorded from above with a B&W mini surveillance camera (infrared sensitivity, acquisition rate of 30 fps, Henelec 300B) connected to a laptop computer using video recording software (Pinnacle Studio 12, http://www.pinnaclesys.com/). Videos of the test fish were analyzed using a commercial video tracking software (Ethovision XT 11.0, Noldus Inc., The Netherlands). A region of interest (ROI) corresponding to the mean body length of an adult zebrafish (10% of the tank), plus the width of the tank was used. The percentage of time fish spent in each ROI (ROI1 and ROI2) was used to calculate the preference score for that stimulus [time in ROI1 / (time in ROI1 + time in ROI2)] and to calculate stimuli exploratory score [(time in ROI1 + time in ROI2) / Total time].

### Statistics

Normality of the data was tested with both the Shapiro-Wilk normality test and D´Agostino & Pearson omnibus normality test. The homogeneity of variance was tested using the Brown-Forsythe test. When parametric assumptions were verified, on raw or transformed data, we used parametric tests. When, even after transforming data, parametric assumptions were not met, non-parametric statistics were used. To assess the preference of the fish for the two-presented stimuli, the percent of cumulative time fish spent in each ROI was compared with paired t-tests or Wilcoxon matched-pairs signed-ranks. The comparisons of the preference and exploratory scores between different experiments were performed with Mann Whitney test or unpaired t-test, or one-way ANOVA or Kruskal-Wallis followed by post-hoc tests to identify the differences between treatments. For transcriptional activation assay, statistical significances were determined by one-way ANOVA test.

Effect sizes were reported and reference effect size values (small for d > 0.2, medium for d > 0.5, and large for d > 0.8) used to interpret the mean difference of the effect (Cohen, 1988).

Statistical analyses were performed using Graphpad Prism software (version 6.0c). For all tests the significance level used was p < 0.05.

## Supplementary information

Supplementary Information.

Supplementary Information2.

Supplementary Information3.

## Data Availability

The datasets generated during and/or analyzed during the current study are available from the corresponding author on reasonable request.
