# Supplementary material for: Perceptual mechanisms of social affiliation in zebrafish
Source: Sci Rep. 2020 Feb 27;10:3642. doi: 10.1038/s41598-020-60154-8 (PMC7046791; doi:10.1038/s41598-020-60154-8)
Supplement: Supplementary file 1 — Supplementary Information. [file 41598_2020_60154_MOESM1_ESM.pdf]

**Supplementary Information file**

# Perceptual mechanisms of social affiliation in zebrafish

**Authors:**

Ana Rita Nunes[1*], Leonor Carreira [1], Savani Anbalagan[2,4], Janna Blechman[2], Gil Levkowitz[2], Rui F. Oliveira[1,3*]

**Affiliations:**

1- Gulbenkian Institute of Science, Oeiras, Portugal

2- Weizmann Institute of Science, Rehovot, Israel

3- ISPA - Instituto Universitário, Portugal

4-Current address: ReMedy-International Research Agenda Unit, Centre of New Technologies, University of Warsaw, 02-097 Warsaw, Poland; Laboratory of Glial Biology, Centre of New Technologies, University of Warsaw, 02-097 Warsaw, Poland.

## Supplementary Methods

### Shoal preference

To test the preference towards a real shoal, a focal fish was placed in a central compartment (30x15x10 cm) of a three-tank behavioural setup. Two compartments were placed at each lateral side of the central tank, blocked by an opaque partition. One compartment contained a shoal of fish (social cue, 2 females and 2 males) while the other was kept empty. A one-way (unidirectional) mirror was placed in-between the central compartment and the lateral compartments to prevent interactions between the shoal and focal fish. In this way, the focal fish could see the shoal without being seen. Illumination was placed over the shoal and empty compartments, creating the lightening conditions required for the mirror effect. To avoid side bias the stimuli were randomly assigned to these two compartments. After an acclimatization period of 10 min, the opaque partitions were lifted and the focal fish allowed to explore the stimuli, while its behaviour was video-recorded.

To test the preference towards a video of a shoal, a focal fish was placed in the videoplayback setup described in the methods section of this manuscript (see methods section for videoplayback behavioural protocol). During the

preference phase, one of the LCD screens displayed a video of a shoal and the other, one tank containing water.

The time spent by the focal fish near (within one body length) each compartment was quantified and used to calculate the social preference score (%Time in shoal ROI/(%Time in Shoal ROI + %Time in Empty ROI).

**Statistical analysis**

For non-parametric data the following transformations were made:

- Log (x) for the following variables: % cumulative time in FISH BM vs. FISH NBM; % cumulative time in FISH NBM vs. DOT BM; % cumulative time in acceleration vs. non-acceleration cues (6 min trial); % cumulative time in elongated shape vs. dot, both with acceleration cues; % cumulative time in start from rest vs. ambiguous (6 min trial); stimuli exploration score of $oxtr^{+,+}$ and $oxtr^{-/-}$ presented to static fish vs. static dot; stimuli exploration score of $oxtr^{+,+}$ and $oxtr^{-/-}$ presented to FISH BM vs. FISH NBM; mean speed of $oxtr^{+,+}$ vs. $oxtr^{-/-}$ presented to acceleration vs. non-acceleration stimuli;
- Log (x+1) for the % cumulative time in start from rest vs. ambiguous (2 min trial);
- Arcsine for the preference score of $oxtr^{+,+}$ and $oxtr^{-/-}$ when presented to DOT BM vs. DOT NBM.
- Square root for the following variables: % cumulative time in DOT BM vs. DOT NBM; % cumulative time in acceleration vs. non-acceleration cues (2 min trial).

**Supplementary Figure and video legends**

**Supplementary Figure S1- Biological Shape promotes zebrafish social affiliative behaviors**

**(a-d) Validation of the videoplayback system. (a)** Fish were allowed to choose between a real shoal vs. an empty compartment. % Cumulative time fish spent near the shoal vs. empty tank. **(b)** Fish were allowed to choose between a video of a shoal vs. a video of an empty compartment. % Cumulative time fish spent near the video of a shoal vs. a video of an empty tank. **(c)** Comparison of the preference score towards a shoal in the experiments described above. **(d)** Conspecific form promotes zebrafish social

affiliative behavior. Zebrafish were allowed to choose between static images of a fish (FISH, dark grey) and a dot (DOT, light grey). 1 Minute - time bins of % time fish spent near the stimuli. **(e)** Fish were allowed to choose between a fish with biological motion (FISH BM, dark grey) vs. a dot with biological motion (DOT BM, light grey). 1 Minute - time bins of % time fish spent near the stimuli. **(f)** Fish were allowed to choose between a fish with non- biological motion (FISH NBM, dark grey) vs. a dot with non-biological motion (DOT NBM, light grey). 1 Minute - time bins of % time fish spent near the stimuli. Error bars and shaded regions indicate SEM. ****P<0.0001.

**Supplementary Figure S2- Biological motion promotes zebrafish social affiliative behaviors. (a)** Fish were allowed to choose between a dot with biological motion (DOT BM, dark grey) vs. a dot with non- biological motion (DOT NBM, light grey). 1 Minute - time bins of % time fish spent near the stimuli. **(b)** Fish were allowed to choose between a fish with biological motion (FISH BM, dark grey) vs. an image of a fish moving with non-biological motion (FISH NBM, light grey). 1 Minute-time bins of % time fish spent near the stimuli. Shaded regions indicate SEM.

**Supplementary Figure S3- Congruent social stimuli promote social preference. (a)** Fish were allowed to choose between a fish moving with biological motion (FISH BM, dark grey) vs. a dot with non- biological motion (DOT NBM, light grey). 1 Minute - time bins of % time fish spent near the stimuli. **(b)** Fish were allowed to choose between a dot moving with biological motion (DOT BM, dark grey) vs. an image of a fish moving with non-biological motion (FISH NBM, light grey). 1 Minute-time bins of % time fish spent near the stimuli.
Shaded regions indicate SEM.

**Supplementary Figure S4- Zebrafish perceives elementary cues of biological motion and shape enhances this preference. (a) –** Fish were allowed to choose between a dot moving with speed changes (ACCEL., dark grey) vs. a dot with constant speed (NON ACCEL., light grey). 1 Minute - time bins of % time fish spent near the stimuli. Depict of the % cumulative time in ROI during the first 2 min of the trial is shown. **(b)** Fish were allowed to

choose between a dot moving with a single speed change (S.ACCEL., dark grey) vs. multiple speed changes (M.ACCEL., light grey). 1 Minute-time bins of % time fish spent near the stimuli. **(c)** Fish were allowed to choose between an image of a fish moving with a single speed change (FISH ACCEL., dark grey) vs. an image of fish moving with constant speed (FISH NON ACCEL., light grey). 1 Minute-time bins of % time fish spent near the stimuli. **(d)** Comparison of the stimuli exploration score between dot and image of a fish moving with speed changes vs. constant speed. **(e)** Comparison of the preference score between dot and image of a fish moving with speed changes vs. constant speed. **(f)** Shape enhances attraction to acceleration cues. Fish were allowed to choose between two identical dots with the same speed change. **(g)** % Cumulative time fish spent in each dot with speed changes. **(h)** 1-Minute time bins of % time fish spent near the stimuli. **(i)** Fish were allowed to choose between an elongated shape vs. a dot, both with speed changes. **(j)** % Cumulative time fish spent near elongated shape (black dots) and dot (grey squares). **(k)** 1-Minute time bins of % time fish spent near elongated shape (dark grey) and dot (light grey). **(l)** Fish were allowed to choose between an image of a fish vs. a dot, both with the same speed changes. **(m)** % Cumulative time fish spent near the fish image (black dots) and dot (grey squares). **(n)** 1-Minute time bins of % time fish spent near the fish image (dark grey) and dot (light grey). **(o)** Fish were allowed to choose between a dot that start from rest vs. an ambiguous dot. **(p)** 1-Minute time bins of % time fish spent near the start from rest dot (dark grey) vs. an ambiguous dot (light grey).

Error bars and shaded regions indicate SEM. $*P<0.5$, $***P<0.001$.

**Supplementary Figure S5- Oxytocin signaling is differently involved in the perception of biological motion and conspecific form. (a)** Fish were allowed to choose between static images of a fish vs. a dot and respective **(b)** 1-minute time bins of stimuli exploration score for *oxtr*$^{(+/+)}$ (grey) and *oxtr*$^{(-/-)}$ (cyan) and **(c)** 1-minute time bins of preference score for *oxtr*$^{(+/+)}$ (grey) and *oxtr*$^{(-/-)}$ (cyan). **(d)** Fish were allowed to choose between a dot moving with BM (DOT BM) vs. a dot with non-biological motion (DOT NBM) and respective **(e)** 1-minute time bins of stimuli exploration score for *oxtr*$^{(+/+)}$ (grey) and *oxtr*$^{(-/-)}$

(cyan) and **(f)** 1-minute time bins of preference score for *oxtr*$^{(+/+)}$ (grey) and *oxtr*$^{(-/-)}$ (cyan). **(g)** Fish were allowed to choose between a fish moving with BM (FISH BM) vs. an image of a fish moving with non-biological motion (FISH NBM) and respective **(h)** 1-minute time bins of stimuli exploration score for *oxtr*$^{(+/+)}$ (grey) and *oxtr*$^{(-/-)}$ (cyan) and **(i)** 1-minute time bins of preference score for *oxtr*$^{(+/+)}$ (grey) and *oxtr*$^{(-/-)}$ (cyan). **(j)** Fish were allowed to choose between a fish moving with BM (FISH BM) vs. a dot with non-biological motion (DOT NBM) and respective **(k)** 1-minute time bins of stimuli exploration score for *oxtr*$^{(+/+)}$ (grey) and *oxtr*$^{(-/-)}$ (cyan) and **(l)** 1-minute time bins of preference score for *oxtr*$^{(+/+)}$ (grey) and *oxtr*$^{(-/-)}$ (cyan). **(m)** Fish were allowed to choose between a dot moving with speed changes (ACCEL) vs. a dot with constant speed (NON ACCEL.) and respective **(n)** 1-minute time bins of stimuli exploration score for *oxtr*$^{(+/+)}$ (grey) and *oxtr*$^{(-/-)}$ (cyan), **(o)** 1-minute time bins of preference score for *oxtr*$^{(+/+)}$ (grey) and *oxtr*$^{(-/-)}$ (cyan) and **(p)** mean speed for *oxtr*$^{(+/+)}$ (grey) and *oxtr*$^{(-/-)}$ (cyan). **(q)** Fish were allowed to choose between a dot that start from rest (START FROM REST) vs. an ambiguous dot (AMBIGUOUS) and respective **(r)** 1-minute time bins of stimuli exploration score for *oxtr*$^{(+/+)}$ (grey) and *oxtr*$^{(-/-)}$ (cyan), **(s)** 1-minute time bins of preference score for *oxtr*$^{(+/+)}$ (grey) and *oxtr*$^{(-/-)}$ (cyan) and **(t)** mean speed for *oxtr*$^{(+/+)}$ (grey) and *oxtr*$^{(-/-)}$ (cyan). Error bars and shaded regions indicate SEM. $^{**}P<0.01$.

**Supplementary Table 1-** Oligonucleotide primers used to amplify DNA templates for *oxtr* and mut *oxtr* amplification reactions.

**Supplementary video1** – Sample of the video playback stimuli presented in all experiments except the ones reported in Fig.4. An image of an empty tank was shown during the acclimatization phase. During the preference phase, each LCD screens showed a different stimulus, in the pair of combinations reported in the figures.

**Supplementary video2** – Sample of the video playback stimuli presented in the experiments reported in Fig.4 and Fig.S5. During the preference phase, each LCD screens showed a different stimulus, in the pair of combinations reported in the figures.

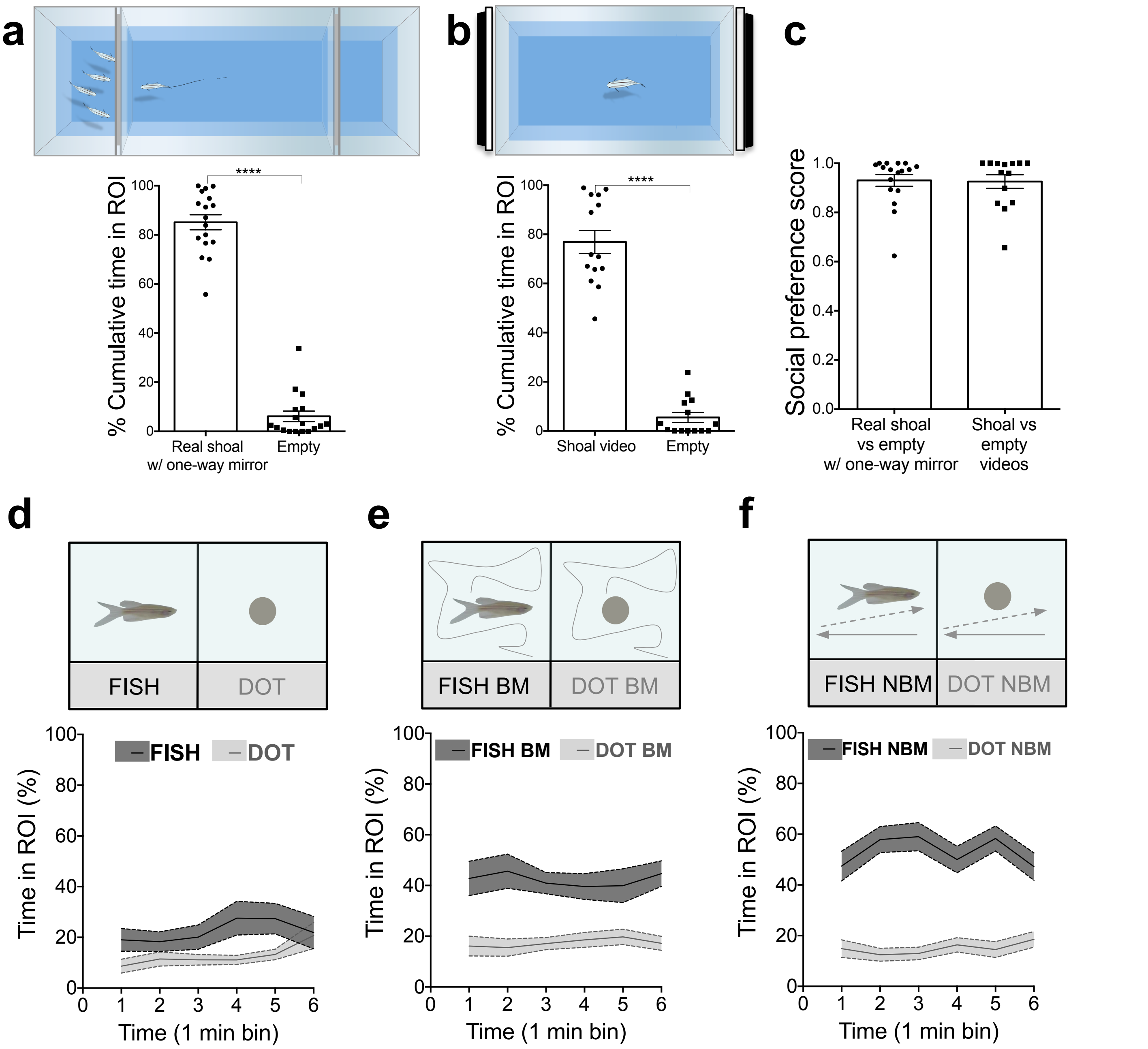


**Nunes A.R. *et al*, Supplementary Fig. S1**

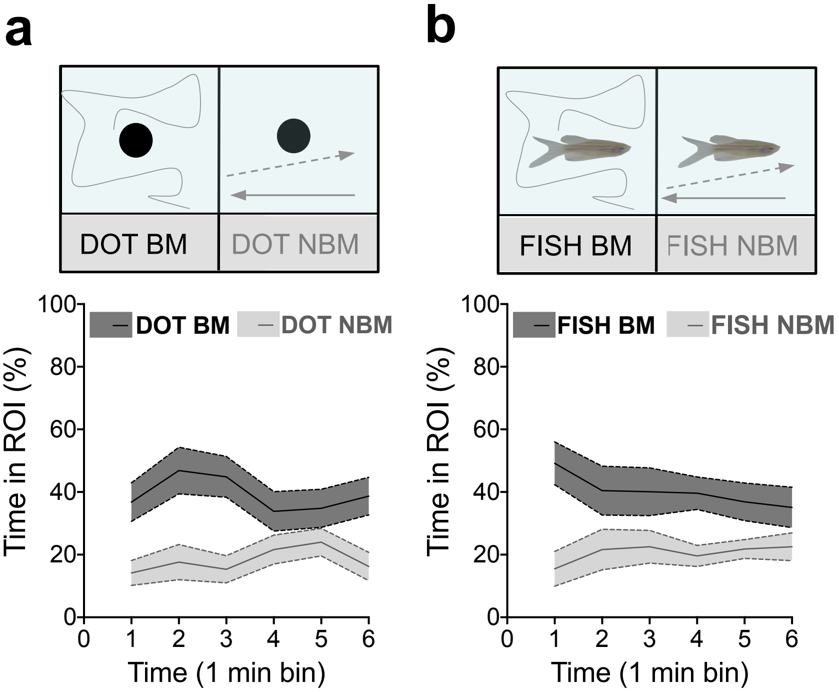


Nunes A.R. *et al*, Supplementary Fig. S2

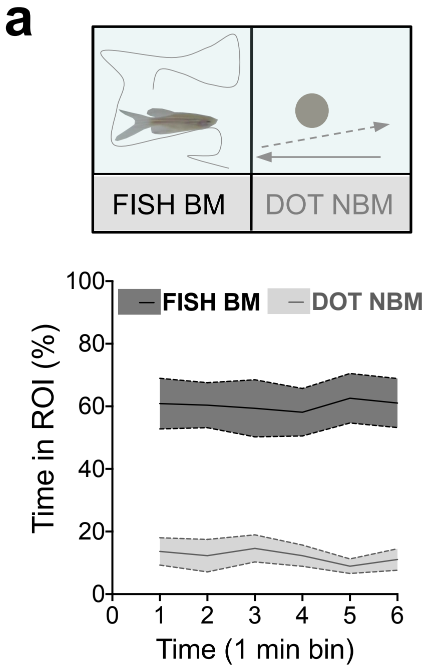


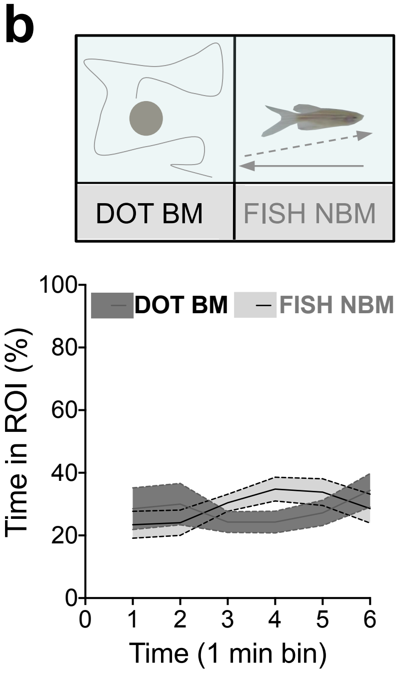


**Nunes A.R. *et al*, Supplementary Fig. S3**

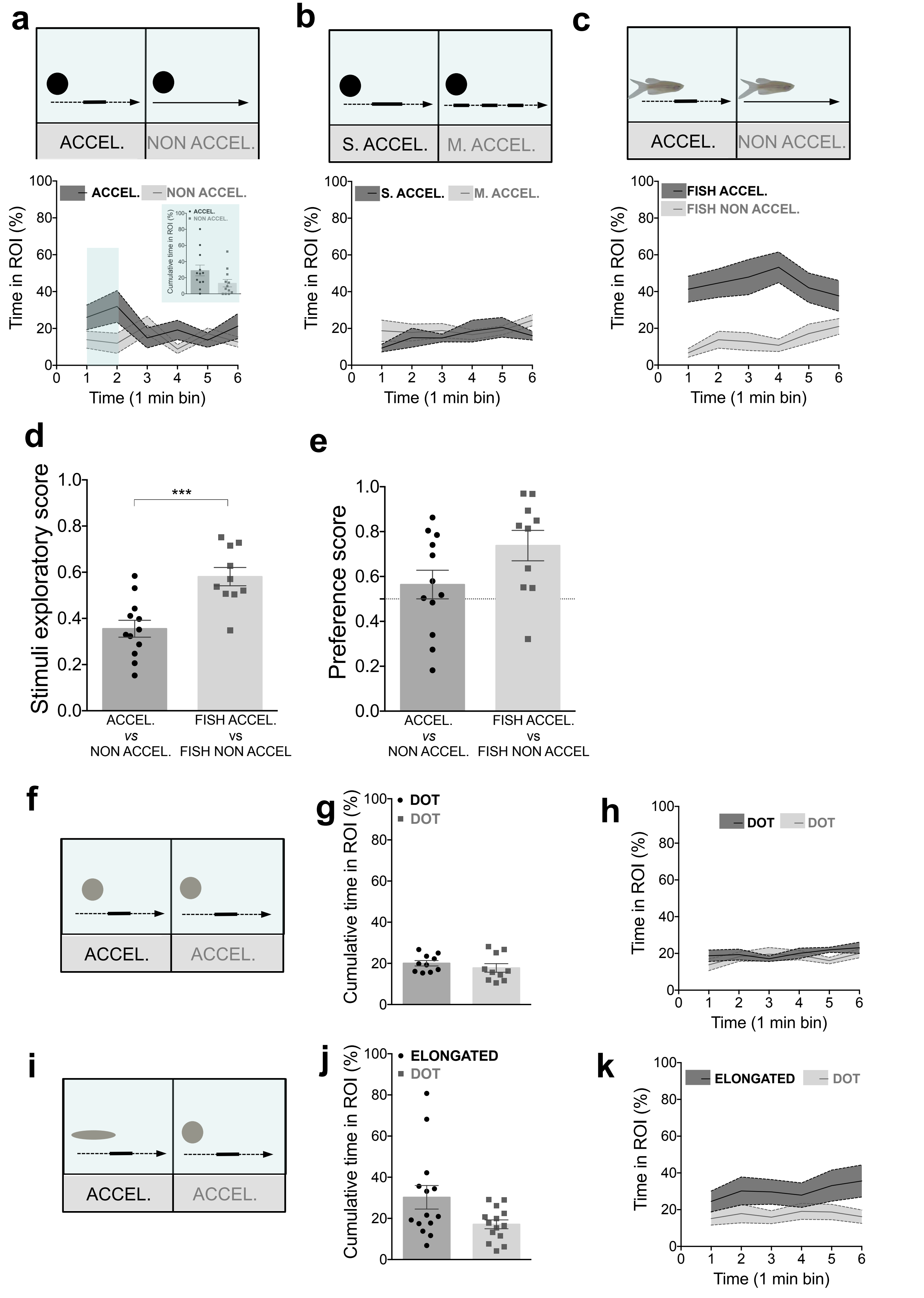


**Nunes A.R. *et al*, Supplementary Fig. S4**

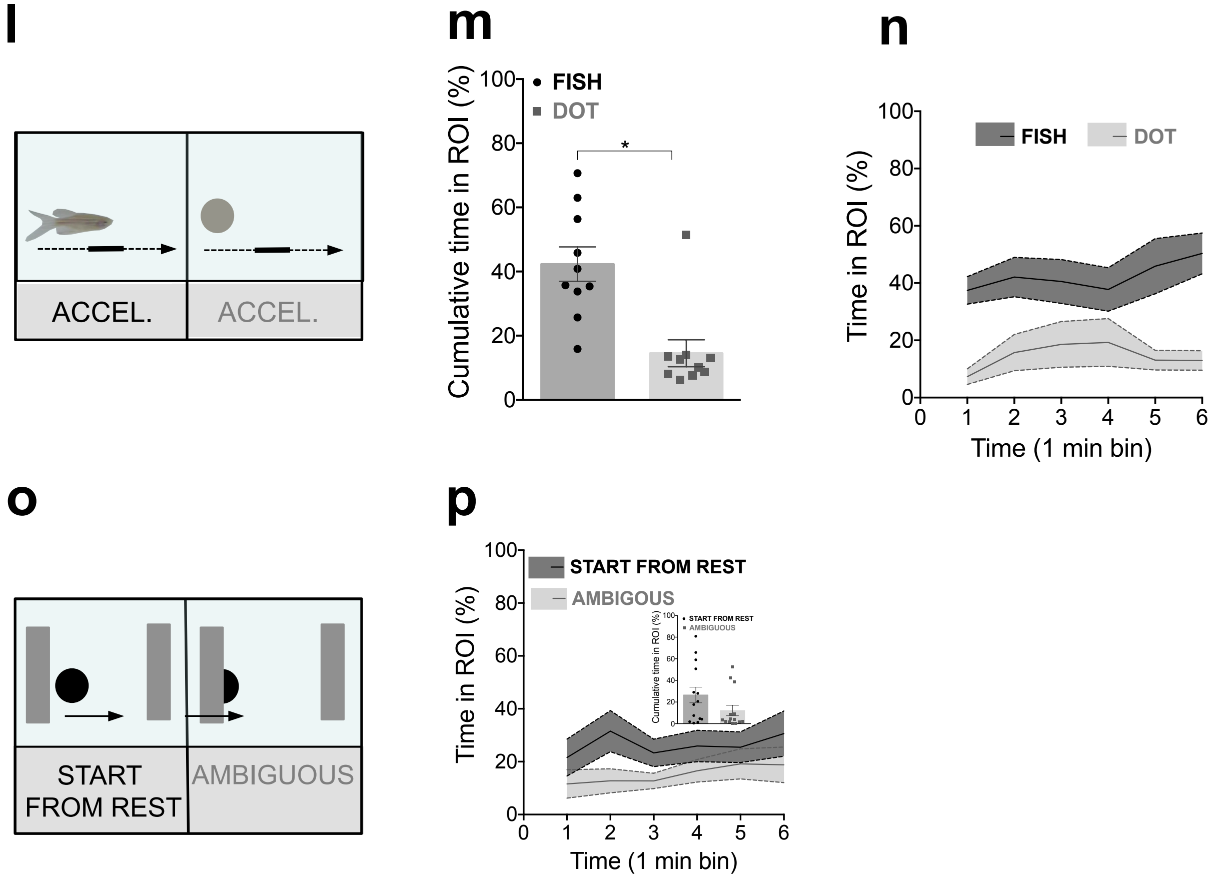


**Nunes A.R. *et al*, Supplementary Fig. S4 (cont.)**

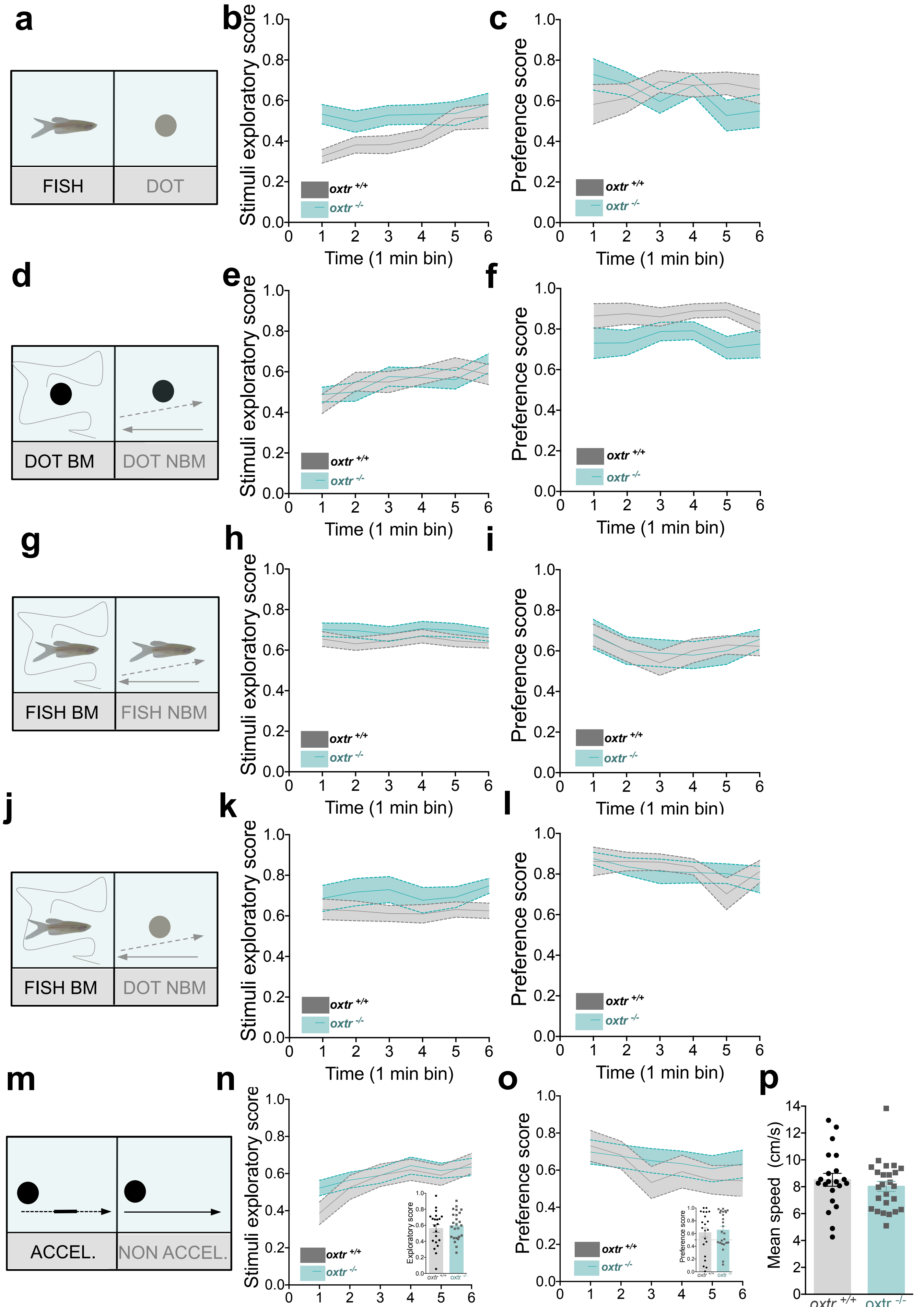


Nunes A.R. *et al*, Supplementary Fig. S5

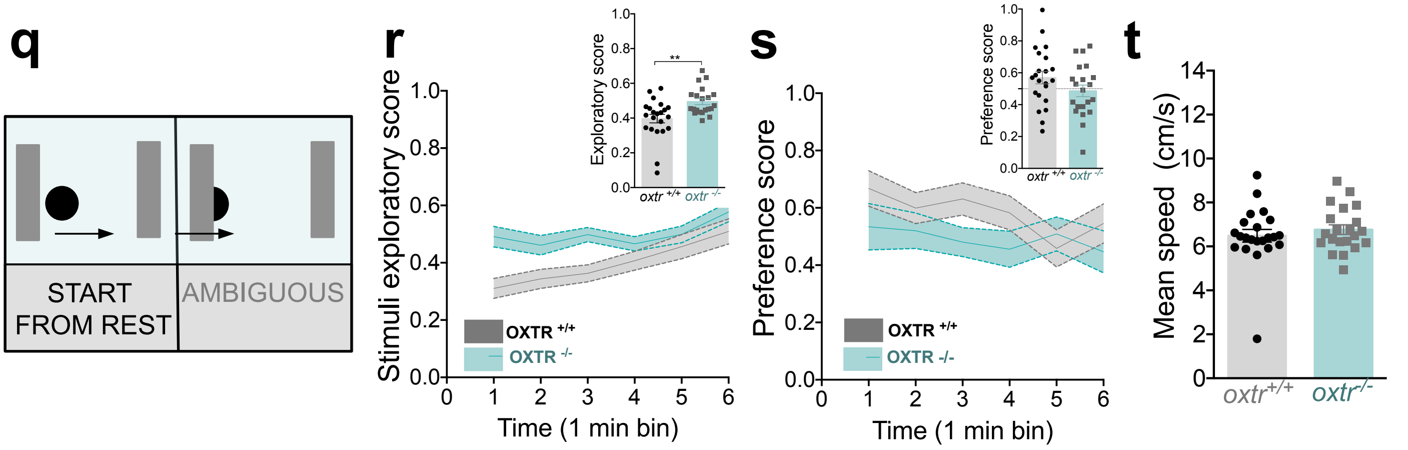


**Nunes A.R. *et al*, Supplementary Fig. S5 (cont.)**

**Nunes A.R. *et al*, Supplementary table 1**

| | |
|---|---|
| oxtr_atg (EcoRI) | AGTCGAATTCATGGAGGACATCTTCAAGGATCA |
| oxtr_stop (XbaI) | AGTCTCTAGATTATGTGATGGAGGTTTGGGTGAT |
| oxtr_mut 5’ | ACTTTTGGTCCTTTATGAGTCATCCA |
| oxtr_mut 3’ | TGGATGACTCATAAAGGACCAAAAGT |
| oxtr_EcoNI | TCCCCAGCAATCATAGACTCCT |
